# Cytochrome P450 CYP1A1: wider roles in cancer progression and prevention

**DOI:** 10.1186/1471-2407-9-187

**Published:** 2009-06-16

**Authors:** Vasilis P Androutsopoulos, Aristidis M Tsatsakis, Demetrios A Spandidos

**Affiliations:** 1 Department of Medicine, Division of Forensic Sciences and Toxicology, University of Crete, Crete, Greece; 2 Department of Medicine, Division of Clinical Virology, University of Crete, Crete, Greece

## Abstract

CYP1A1 is one of the main cytochrome P450 enzymes, examined extensively for its capacity to activate compounds with carcinogenic properties. Continuous exposure to inhalation chemicals and environmental carcinogens is thought to increase the level of CYP1A1 expression in extrahepatic tissues, through the aryl hydrocarbon receptor (AhR). Although the latter has long been recognized as a ligand-induced transcription factor, which is responsible for the xenobiotic activating pathway of several phase I and phase II metabolizing enzymes, recent evidence suggests that the AhR is involved in various cell signaling pathways critical to cell cycle regulation and normal homeostasis. Disregulation of these pathways is implicated in tumor progression. In addition, it is becoming increasingly evident that CYP1A1 plays an important role in the detoxication of environmental carcinogens, as well as in the metabolic activation of dietary compounds with cancer preventative activity. Ultimately the contribution of CYP1A1 to cancer progression or prevention may depend on the balance of procarcinogen activation/detoxication and dietary natural product extrahepatic metabolism.

## Background

Cytochrome P450s are haem-containing enzymes, which catalyze various Phase I metabolism reactions, such as C-, N- and S- oxidation and dealkylation. Cytochrome P450 CYP1A1 is one of the three members of the CYP1 family, which is found mainly in extrahepatic tissues and participates in the metabolism of a vast number of xenobiotics, as well as a small number of endogenous substrates. Among the different reactions catalyzed by CYP1A1, hydroxylation at a vacant position of an aromatic ring is considered to be the hallmark for the initiation of carcinogenesis, through the formation of highly reactive conversion products that can cause oncogenic mutations in experimental animals and humans [1,2]. The transcriptional activation of the *CYP1A1 *gene is mediated by the binding of environmental pollutants and inhalation chemicals, notably substrates of the CYP1A1 enzyme, to the cytosolic receptor AhR and is also mediated by its translocation to the nucleus and subsequent formation of a dimer, which interacts with the corresponding xenobiotic response elements to activate transcription [3]. Although the xenobiotic-activating pathway of AhR has been well established for a large number of exogenous ligands, the receptor has been shown to participate in important developmental and cell-regulatory processes, except foreign compound metabolism. These functions coexist with the well-characterized toxicological roles of the receptor. As a result the exact function of CYP1A1 appears to be a lot more complex than initially thought. Recent *in vivo *investigations suggest that CYP1A1 may function as a carcinogen-detoxication enzyme, whereas the paradoxical activation of natural dietary compounds with chemopreventative activity provides further insight into the cancer-protecting role of this enzyme.

In this review, a comprehensive summary of the carcinogen-activating role of CYP1A1 is presented, in terms of substrate specificity, mechanisms of carcinogen activation, polymorphisms and extrahepatic expression. In addition, experimental evidence of the interaction of AhR with various biological pathways, including cell cycle control, apoptosis, mitogen-activated protein kinases, estrogen receptor, glucocorticoid receptor and hypoxia signaling are addressed. Finally, recent findings with transgenic animals and *in vitro *pharmacological evidence, which point towards a cancer-protecting role of this enzyme, are presented.

## Discussion

### Mechanism of activation of procarcinogens by CYP1A1

The deleterious effects of most of the chemical carcinogens encountered in the environment are attributed to metabolic activation by cytochrome P450s to highly reactive conversion products. It has been proven that such reactive metabolites cause carcinogenicity in experimental animals and humans whereas their corresponding parent compounds are chemically inactive [4,5]. Cytochrome P450 CYP1A1 is one of the more significant P450 enzymes involved in this process. CYP1A1 metabolizes carcinogens to epoxide intermediates, which are further activated to diol epoxides by the enzyme epoxide hydrolase. The widely accepted paradigm used to demonstrate this process is the activation of the carcinogen Benzo[*a*]pyrene B[*a*]P.

**Figure 1:**
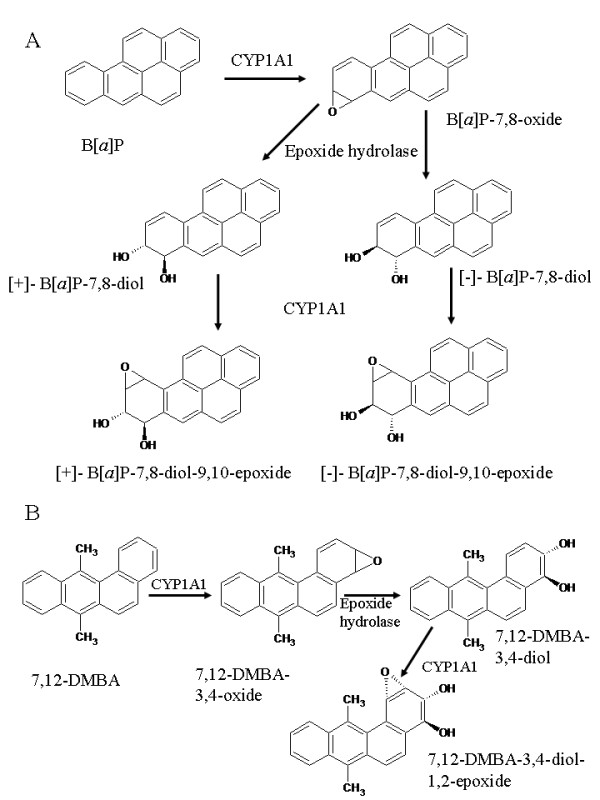
**Metabolic activation of (A) B[*a*]P and (B) 7,12-DMBA to the carcinogenic metabolites B[*a*]P-7,8-diol, B[*a*]P-7,8-diol-9,10-epoxide and 7,12-DMBA-3,4-diol, 7,12-DMBA-3,4-diol-1,2-epoxide, respectively, by CYP1A1 and epoxide hydrolase **[6].

The metabolic fate of the prototype carcinogen B[*a*]P was extensively studied in the mid-1970s in humans [4]. It was initially thought that the metabolite B[*a*]P-4,5-epoxide, the so-called K-region epoxide, was the ultimate carcinogen. However subsequent investigations clearly demonstrated that B[*a*]P-7,8-diol-9,10-epoxides, referred to as bay region epoxides, were highly reactive towards DNA and thus were classified as the ultimate carcinogenic metabolites of B[*a*]P. The exact mechanism of metabolic activation involves the oxidation of B[*a*]P to B[*a*]P-7,8-oxide, and subsequent hydrolysis to B[*a*]P-7,8-diol and the two enantiomers (+)-B[*a*]P-7,8-diol and (-)-B[*a*]P-7,8-diol [6]. A final oxidation of each of these metabolites produces four diol epoxides, which are highly mutagenic in Ames *Salmonella *tester strains and Chinese Hamster V-79 cells. Additionally the epoxides are denoted as bay region epoxides due to their ability to cause oncogenic mutations in specific parts of the DNA [2,6,7]. The metabolite (+)-B[*a*]P-7,8-diol-9,10-epoxide-2 was identified as the most reactive of the four metabolites in producing tumors in newborn mice. This metabolite was considered to be the ultimate carcinogenic conversion product of B[*a*]P, because its level of carcinogenicity paralleled that of B[*a*]P and (-)-B[*a*]P-7,8-diol. The structures of B[*a*]P and its metabolites are shown in Figure 1A.

With the exception of the hydrolysis step catalyzed by epoxide hydrolase, the oxidation reactions of B[*a*]P activation are promoted by cytochrome P450s. Among the different families, CYP1A1 and CYP1B1 exhibit the highest catalytic specificity towards B[*a*]P, as shown by *in vitro *experiments with recombinant human P450 enzymes from *E. Coli *and *Trichoplusia ni *cells [6]. Other PAHs have been investigated for their carcinogenic action and found to follow the same metabolic activation pattern as B[*a*]P. The carcinogen 7,12-Dimethyl benzanthracene (7,12-DMBA) is oxidized to 7,12-DMBA-3,4-oxide by CYPs, further hydrolyzed to its corresponding diol and finally oxidized by CYPs to 7,12-DMBA-3,4-oxide-diol-1,2-epoxide, which is the ultimate carcinogen (Figure 1B) [6]. Carcinogenic compounds which fall in the PAH category and are considered to follow the bay region activation theory include benz[*a*]anthracene, benzo[*b*]fluoranthrene, benzo[*c*]phenanthrene, chrysene, benzo[*g*]chrysene and 5,6-dimethylchrysene. The above-mentioned PAHs showed selective *in vitro *metabolism towards human recombinant CYP1A1 and CYP1B1 and were capable of inducing DNA-modifying products in the Salmonella typhimurium NM2009 tester strain [8]. CYP1A1 is further involved in the activation of aflatoxin B1, a carcinogenic mycotoxin present in foodstuffs, to its corresponding 8,9-epoxide in rabbit lung and liver. Further *in vivo *investigations have shown that CYP1A1 is involved in B[*a*]P-induced carcinogenesis in mice which were positive for the aryl hydrocarbon receptor AhR (+/+) [6,9,10]. Studies in this transgenic strain demonstrated an increase in CYP1A1 expression in both liver and lung, following treatment of PAHs, such as 5-methylchrysene and 7,12-DMBA. Hence, the carcinogenic potential of CYP1A1 in the activation of PAHs has been well documented both *in vitro *and *in vivo*.

CYP1A1 was thought to be uniquely responsible for PAH activation, until the early 1990s, when CYP1B1 was identified. Generally the substrate specificities of the two enzymes towards various pro-carcinogens and pro-mutagens are found to be very similar, even though recombinant human CYP1A1 and CYP1B1 differ in their region and stereochemical selectivity for the activation of certain compounds e.g. DB[a, l]P [6,11,12].

**Figure 2:**
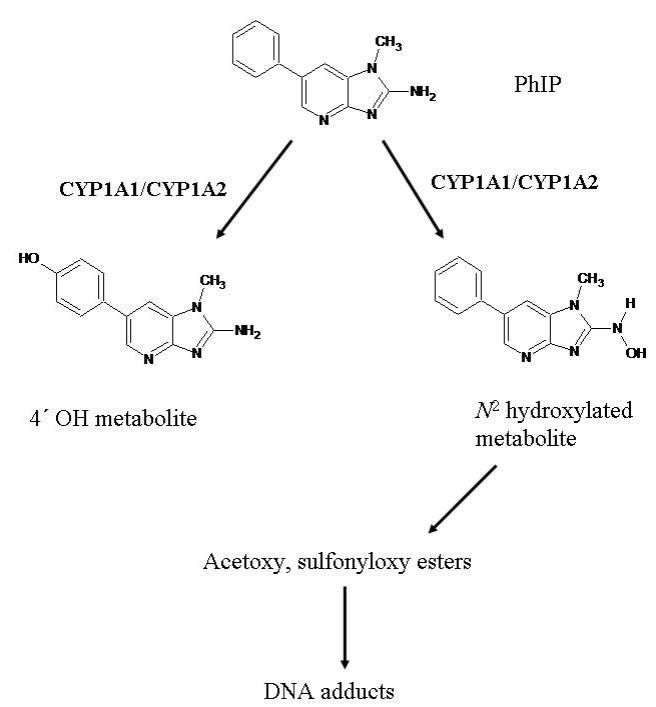
**Metabolic activation of PhIP to the carcinogenic metabolite *N*^2^-hydroxy PhIP by CYP1A enzymes and epoxide hydrolase **[14].

The metabolic activation of heterocyclic amines is also catalyzed by CYP1A1. PhIP or 2-Amino-1-methyl-6-phenylimidazo[4,5-b]pyridine is the most abundant heterocyclic amine in food, which is a product of cooked meat and fish. The hydroxylation of PhIP at position *N*^2^- is considered as the initiation step of PhIP-induced carcinogenesis [13]. Hydroxylation at position *N*^2^, catalyzed by CYPs, is followed by esterification with N-acetyltransferase or sulfotransferase to produce the corresponding esters *N*^2^-acetoxy-PhIP and *N*^2^-sulfonyloxy-PhIP [14]. These esters form covalent bonds with DNA to yield *N*^2^-(2-deoxyguanosin-8-)-PhIP, and with proteins to the metabolite 5-hydroxy-PhIP, a degradation product occurring spontaneously. The chemical structures of PhIP and metabolites are shown in Figure 2.

PhIP-DNA adducts have been detected in various tissues of rats and mice, as well as in the colon of humans [15]. Although CYP1A2 is a significant isoform involved in PhIP activation in the liver, CYP1A1 plays an equally important role in this process in extrahepatic tissues, such as the lung. Early studies with knockout mice have shown that PhIP-DNA adducts were detected in extrahepatic tissues of Cyp1a2 null mice, implying a role of CYP1A1 in PhIP metabolic activation [16]. A recent study by Gonzalez demonstrated that the *N*^2^-hydroxylation of PhIP was increased almost 2-fold in lung homogenates of Cyp1a2 null mice, compared to wild-type mice whereas it reached minimal levels in Cyp1a1 null mice [13]. In addition, PhIP-DNA adducts were significantly higher in lung samples from Cyp1a2 null strains, as opposed to Cyp1a1 null mice and almost equivalent with the amount present in wild-type mice [13]. In the humanized CYP1A2 (CYP1A2_CYP1A1 Cyp1a2 null) transgenic strain it was shown that *N*^2^-hydroxylation was favored over 4'-hydroxylation of PhIP [14]. This implies that in humans oxidation of the exocyclic amino group (*N*^2^-hydroxylation) is the major route of metabolism, followed by glucuronidation, whereas in rats and rodents 4' hydroxylation to 4'-hydroxy PhIP followed by phase II conjugation is the predominant metabolic pathway, which has been shown to be associated with detoxication rather than metabolic activation. However, the humanized CYP1A1 (CYP1A2_CYP1A1 Cyp1a1 null) strain was not examined in this study, in terms of PhIP metabolism [14].

CYP1A1 has also been shown to be involved in the activation of tobacco-related N-nitrosamines, such as NNK, along with CYP1A2 and CYP2A6 [17]. Such compounds induce cancer in experimental animals and their activation step requires hydroxylation of the α-position carbon atom of N-nitroso group, a reaction catalyzed by CYPs [18].

### CYP1A1 induction is mediated by the AhR, a receptor involved in various biological processes

**Figure 3:**
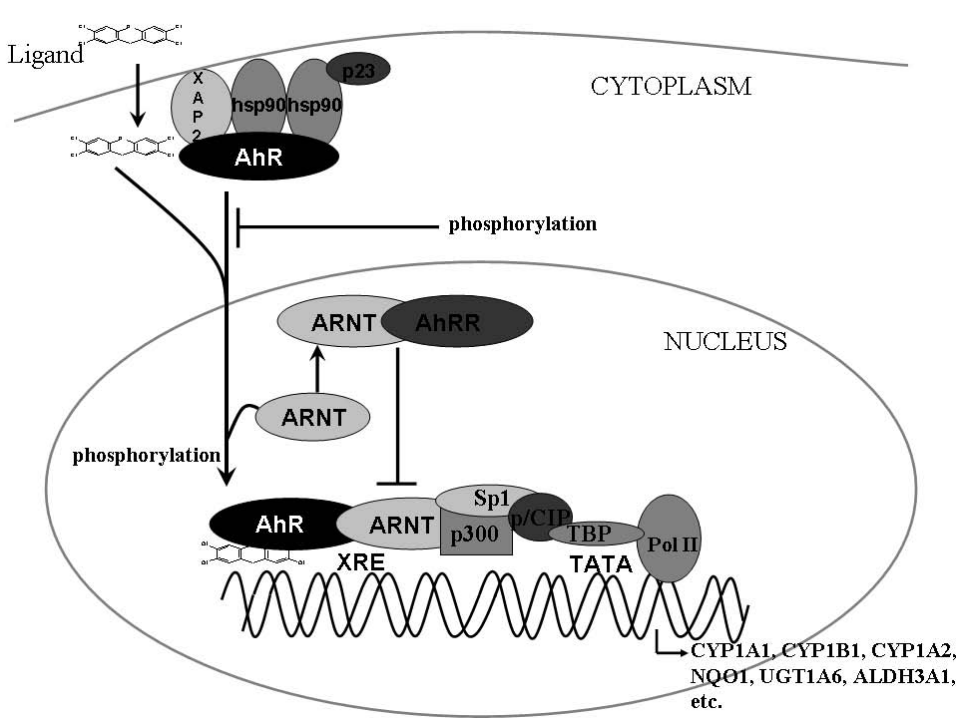
**AhR ligand-mediated activation of phase I and II metabolizing enzyme genes**. The diagram represents a basic model of the molecular events following the entry of an AhR ligand, such as TCDD, in the cell. Upon ligand binding the AhR complex dissociates with XAP2, p23 and HSP90 proteins and translocates to the nucleus. Nuclear import is inhibited by phosphorylation reactions of either Ser-12 or Ser-36 residues of the Nuclear localization signal (NLS), while phosphorylation of phosphotyrosine residues in the carboxy terminal of the AhR is required for the formation of a functional AhR/ARNT complex [19]. Binding of the AhR/ARNT complex to XRE is inhibited by the ARNT/AhRR dimer. Initiation of the transcription of genes encoding for phase I and phase II metabolizing enzymes occurs via the interaction of several transcription factors such as Sp1 and co-activators such as p-300 and p/CIP, which eventually leads to binding with TBP (TATA binding protein) and subsequent recruitment of RNA polymerase II [20-22]. There is a great number of other transcription factors, co-activators and general transcription factors (GTFs) involved in this process, which are not shown for clarity.

The induction of CYP1A1 expression is mediated through a specific cytosolic receptor, the Aryl hydrocarbon receptor or AhR. AhR exists as part of a cytosolic protein complex, which consists of two Hsp-90 heat-shock proteins, a Hsp-90-interacting co-chaperone p23 and an immunophillin-like protein XAP2 or AIP [19]. In the presence of an exogenous ligand such as B[*a*]P or the industrial byproduct 2,3,7,8-tetrachlorodibenzo-*p*-dioxin (TCDD) the receptor complex translocates to the nucleus, where it heterodimerizes with another protein, the aryl hydrocarbon nuclear translocator or ARNT (Figure 3). This heterodimer binds to consensus regulatory sequences termed AhREs (Aryl hydrocarbon response elements) XREs (Xenobiotic response elements) or DREs (Dioxin response elements), located in the promoter region of AhR target genes such as CYP1A1 and CYP1A2 and initiates their transcription by recruiting RNA polymerase II (Figure 3) [19]. The transcription of CYP1A1 is inhibited by the AhR-related factor Aryl hydrocarbon receptor repressor or AhRR, which localizes in the nucleus in the form of a dimeric protein along with ARNT (Figure 3). The AhRR/ARNT heterodimer acts as a repressor both by stopping transcription initiated at the XREs and by competing with AhR for heterodimer formation with ARNT. All AhR, ARNT and AhRR are members of the bHLH (basic helix-loop-helix) PAS (Per-ARNT-Sim) family of proteins. The bHLH motif is shared by other transcription factors such as Myc and MyoD and is the protein part essential for DNA binding of the AhR complex [20]. Heterodimerisation of AhR/ARNT is facilitated by interactions between bHLP and PAS domains. Further interactions of the AhR/ARNT heterodimer with transcription factors such as Sp1 and NF-1 are essential to enhance the expression of the *CYP1A1 *gene. Other proteins which possess HAT (Histone Acetyltrasnferase) activity and act as co-activators include SRC-1 (Steroid receptor co-activator), NcoA2 (Nuclear co-activator 2), p/CIP and p300. SRC-1, NcoA2 and p/CIP have been shown to associate with the mouse *Cyp1a1 *enhancer region and to enhance XRE-driven reporter gene transcription [20-22]. Using ChIP analysis, Hatkinson has shown that co-activators such as p300 and p/CIP bind to the enhancer but not to the promoter region of the mouse *Cyp1a1 *gene, following TCDD treatment, whereas RNA polymerase II binds only to the promoter and not to the enhancer region of the same gene [23]. In contrast, Puga and co-workers found that p300 binds both to the enhancer and promoter following B[*a*]P treatment [24]. It is believed that co-activator recruitment, enhances the gene transcription of *CYP1A1 *and aids in the binding of Pol II and transcription factors to the *CYP1A1 *promoter, thus facilitating gene transcription. Nevertheless, it still remains unclear whether participation of co-activator proteins is the same in all tissues, where CYP1A1 is inducible. Further investigation is required for a conclusive report on the molecular events involving co-activator, transcription factor and Pol II recruitment in the transactivation of the *CYP1A1 *gene.

This proposed mechanism of CYP1A1 induction also applies to certain Phase II xenobiotic metabolizing enzymes, such as NQO1, UGT1A6, ALDH3A1 and several glutathione S-transferases, and was initially thought to be the primary role of AhR in the mid 1990s [3]. However, recent discoveries have illuminated a wider function of AhR, than initially thought. It is now generally accepted that the receptor is involved in physiological functions beyond xenobiotic metabolism, such as regulation of cell growth, apoptosis, hypoxia signaling, cell adhesion and matrix metabolism [19,25,26]. Based on accumulating experimental data we broadly categorized the AhR in three important pathways. The first is the extrinsic AhR xenobiotic signaling pathway, which usually requires an exogenous ligand for activation and results in the induction of several Phase I and Phase II metabolizing genes. A second network of pathways involves the interaction of the AhR with various cell-signaling proteins, such as Rb and E2F in the presence or absence of an inducer. The third pathway is the intrinsic AhR pathway, which remains elusive, in terms of exact mechanism of action and is thought to be dependent on an endogenous ligand and play key roles in important physiological and developmental processes. The above mentioned pathways are likely to interact with one another, proving that the exact function of the receptor is a lot more complex, than initially thought.

### Interaction of AhR with protein kinase C and tyrosine kinases

The first line of evidence, which supports a positive interaction between AhR and PKC is derived from early studies in mice, where it was shown that the inhibition of PKC blocks ligand-activated DNA binding of AhR/ARNT heterodimers, leading to a decreased Cyp1 gene expression [27,28]. Previous studies have provided further insight to a more complex signaling mechanism by which the activity of the AhR complex is regulated by PKC. AhR contains a nuclear localization signal (NLS), composed of the amino acid residues 13–16 and 37–39, and a nuclear export signal (NES) [29]. Ikuta showed in 2004 that the ligand-dependent nuclear input of AhR is inhibited by phosphorylation of either Ser12 or Ser36 by PKC. The replacement of Serine residues with Alanine does not affect nuclear translocation, whereas replacement with Asp retains the mutant AhR in the cytoplasm. PKC, however, does not appear to be directly involved in AhR-mediated transcription, as Ala and Asp replacement mutants in *in vitro *luciferase reporter assays had much lower transcriptional activity than the wild type. By analogy to the regulation of NLS, phosphorylation of the Ser 68 residue by p38 of NES, has been demonstrated to activate the export of the receptor from the nucleus [30]. In addition, phosphorylation at the tyrosine residues of the carboxy terminal half of AhR is required for the formation of the functional AhR/ARNT heterodimer, whereas phosphorylation of the Serine residues of HSP90 proteins modulates the formation of a functional cytosolic AhR complex [19]. Thus, it appears that the induction of CYP1A1 is tightly regulated by phosphorylation reactions occurring at the AhR functional domains.

### Cross-talk of MAPK kinases with AhR

MAPK kinases are serine threonine kinases, involved in inflammatory responses, apoptosis, cell growth and further mitogenic and developmental events. It is becoming increasingly accepted that the AhR pathway is linked to an extent with MAPKs. Prototype AhR activators have been shown to have a positive effect on the activation of several MAPKs. For example, the TCDD-induced modulation of epithelial morphology causes the activation of JNK [31]. These dioxin-mediated effects can be mimicked by a constitutive expression of AhR. In addition, ablation of JNK2 and ERK affects TCDD-induced CYP1A1 transactivation by decreasing its expression in mouse thymus and testis [32]. It has been further noted that CYP1A1 and CYP1B1 mRNA and protein expression can be induced in human keratinocytes, after UV exposure. It has been proposed that thryptophan-derived photoreactive products, which are weak agonists of AhR are responsible for this effect, even though the contribution of JNK and p38 activation, which occurs under UV radiation, cannot be entirely dismissed. The induction of ERK and JNK was also noted in A-549 human lung carcinoma and Hepa-1 mouse hepatoma cells, which possess a functional AhR battery, following treatment of TCDD or B[*a*]P [33,34]. In addition, the interaction of AhR with ERK, appears to be critically linked to the function of the receptor, as ERK inhibitors were shown to prolong TCDD-induced AhR degradation. More importantly it has been shown in Hepa1c1c7 cells that ERK is physically associated with AhR, and that the overexpression of ERK1 promoted AhR degradation, suggesting that ERK plays an important role in the proteolysis of the receptor [35]. Moreover, constitutively active MEK1, which is the MAPKK upstream of ERK 1/2, increased the TCDD-mediated induction of CYP1A1 via the AhR [33]. Based on previous investigations regarding cross-talk of the MAPKs with AhR, it can be concluded that exogenous ligands of the receptor contribute to the upregulation of several MAPKs, which in turn exert a positive interaction on the translocation of the AhR complex to the nucleus and subsequently the activation of several Phase I and Phase II metabolizing genes, including *CYP1A1*.

### Role of AhR in cell cycle progression

The effects of AhR in cell cycle progression are distinct, depending on the presence or absence of an exogenous ligand. Although in the absence of a ligand AhR promotes progression of the cell cycle as shown in mouse hepatoma Hepa1c1c7, AhR null MEF cells and HepG2 human hepatoma cells transfected with AhR siRNA, accumulating data strongly suggests that TCDD inhibits cell proliferation [36-40]. TCDD induces cell cycle arrest in normal cells and inhibits the growth of MCF-7 breast adenocarcinoma cells, stimulated by 17-β oestradiol, as well as proliferation of the fish hepatocellular carcinoma PLHC-1 cell line and the androgen-induced LNCap human prostate cancer cell line [41-43]. A delayed G_1 _to S phase transition has been noted in 5L hepatoma cell cultures, following TCDD treatment, which was attributed to induction of the p27^Kip1 ^cell cycle inhibitor. CYP1A1 enzyme activity is thought to act as a negative regulator to the length of AhR activation as 5L cells that were treated simultaneously with serum and the CYP1A1 suicide substrate 1-PP showed prolonged AhR activation and p27^Kip1 ^induction, similar to that of TCDD alone [44,45]. Additional data have shown that AhR blocks the phosphorylation of RB in G_1 _by forming complexes with its hypophosphorylated form [46]. This interaction is supported by two AhR domains, one found in the cyclin D LXCXE motif and the other present within the transactivation domain of the receptor [47]. The end result is the repression of E2F-dependent gene expression, which involves proteins such as cyclin E, CDK2, DNA polymerase α and DHFR and the repression of RB-target genes, as well as the exclusion of co-activator proteins from RB-promoters, suggesting an RB-corepressor function of the AhR [48]. In contrast to these findings, investigations in stably integrated AhR variants in fibroblasts from AhR null mice, show that AhR^+/+ ^proliferate faster than AhR^-/- ^fibroblasts, while the addition of TCDD did not change the rate of proliferation [49]. This indicates a ligand-independent AhR regulation of the cell cycle. In AhR^-/- ^cells cyclin-CDK complexes were downregulated whereas the expression of cell cycle inhibitors was upregulated [49]. In addition, a recent study has shown that constitutively active AhR contributes to basal *CYP1B1 *but not *CYP1A1 *mRNA levels in immortalized and malignant mammary cell lines, whereas AhR hyperactivation by TCDD activates both genes, which implies a contribution of AhR and CYP1B1 prior to tumor formation [50]. These two contradictory bodies of evidence imply the wider function of AhR in the presence and absence of a ligand. It is also important to note that the precise function of AhR in cell proliferation may be different among the various cell or tissue types [19].

### Interaction of AhR with other pathways

AhR has been shown to interact with the glucocorticoid receptor (GR) both *in vitro *and *in vivo *[51-54]. The glucocorticoid dexamethasone has been shown to enhance TCDD-induced expression of CYP1A1, in a rat hepatoma and fish hepatocellular carcinoma cell lines [54,55]. Although many studies have been conducted in rodent models in terms of AhR and GR cross-talk, little information is available for humans [51-53]. Recent evidence from studies in HepG2 cells and primary cultures of human hepatocytes has shown that dexamethasone reduces both basal and inducible CYP1A1 EROD activity [56,57]. Furthermore, dexamethasone was shown to have direct effects on the modulation of TCDD-induced transcriptional activation as well as the degradation of AhR in HepG2 cells. In contrast, experiments conducted in human hepatocytes experiments with the glucocorticoid receptor antagonist RU486 in the presence and absence of dexamethasone, showed a downregulation of basal and TCDD-induced AhR and GR mRNAs and AhR protein [56,57]. These findings show that dexamethasone controls CYP1A1 expression in human hepatocytes and HepG2 cells through interactive regulatory cross-talk between GR and AhR receptors.

Cross-talk of AhR and ARNT with the oestrogen receptor α (ERα) has also been established in a number of different systems [58,59]. TCDD does not bind to ERα, but it inhibits ERα signaling. More importantly ERα plays a significant role in modulating AhR activity, as it has been reported by *in vitro *and *in vivo *studies that treatment of TCDD and E2 results in an increased induction of CYP1A1, compared to TCDD treatment alone [60,61]. ERα has a direct interaction with the *CYP1A1 *promoter, suggesting that it acts as a co-regulator of AhR-mediated transcriptional activation [60]. In human bronchial epithelial cells ERα increased the basal mRNA levels of *CYP1B1 *and the inducible protein levels of *CYP1A1*, thus regulating the expression of these genes at a transcriptional and a translational level, respectively [62]. The interaction of ERβ with AhR and ARNT has also been suggested [63]. Such literature implies that both ERα and ERβ can regulate the expression of carcinogen-metabolizing genes such as *CYP1A1*.

The dimerisation partner of AhR, ARNT is also called HIF-1β (Hypoxia inducible factor 1β). In addition to binding with AhR, ARNT dimerises with the protein HIF-1α to form HIF-1 (Hypoxia inducible factor). HIF-1α is also a member of basic helix-loop-helix (bHLH) Per-ARNT-Sim (PAS) proteins [64,65]. Upon the formation of HIF-1, the induction of transcription of hypoxia-related genes such as VEGF and PDGF is initiated, by the binding of HIF-1α to hypoxia response element (HREs) sequences [66]. Since the sequestration of ARNT is challenged by both AhR and HIF-1α, certain studies have supported the notion that the limiting cellular factor ARNT is shared between two pathways [67-69]. In this sense, reciprocal crosstalk between hypoxia and dioxin signal transduction pathways has been demonstrated to occur *in vitro *and *in vivo *[67]. Hypoxia may downregulate the expression of AhR, and subsequently CYP1A1, as dioxin upregulates the expression of erythropoietin via AhR-ARNT binding to DREs upstream of the transcriptional start site [67]. Increased oxygen supplementation, or hyperoxia has been shown to significantly induce CYP1A1 mRNA, protein and activity in human lung cell lines by means of an AhR-dependent mechanism [70].

### CYP1A1 expression

CYP1A1 is believed to be the primary extrahepatic enzyme involved in the metabolism of carcinogens. Consequently, numerous studies have investigated the expression patterns of CYP1A1 in extrahepatic tissues, which are largely exposed to environmental carcinogens, such as the lung. CYP1A1 has been detected in lung microsomes from human subjects by Western immunoblotting, although the expression was rather weak [71], while EROD activity assays demonstrated active CYP1A1 (range 7–31 nmol/mg/protein/min). This finding has been supported by similar results from different research groups. CYP1A1 mRNA was detected in lung specimens from 27 subjects by semi-quantitative RTPCR in the presence and absence of prototypical and atypical inducers, such as TCDD and pyridine, nicotine and omeprazole respectively [72]. A previous study from the same research group reported on the expression of CYP1A1 to mRNA, the protein and activity levels for some lung specimens, even though considerable variability was noticed in the levels of proteins and transcripts [73]. CYP1A1 expression has further been reported in 40 out of 107 human lung adenocarcinomas and 21 out of 57 mixed bronchioalveolar carcinomas by immunohistochemistry, whereas the expression of both AhR and CYP1A1 was associated with smoking in lung adenocarcinoma patients [74]. Tobacco smoking was shown to be associated with *CYP1A1 *methylation in the lung in another study, as lung samples from active smokers which lack methylation of the CYP1A1 promoter exhibited slightly higher pulmonary EROD activity, in the regression models for age and daily consumption of tobacco [75]. In addition, the expression of CYP1A1 and AhR in small-cell lung carcinoma has been proposed as a putative diagnostic marker and has also been correlated with a history of cigarette smoking [76,77].

Based on these observations the exact function of CYP1A1 in extrahepatic tissues, such as the lung remains unknown. Most studies that have examined the CYP1A1 expression profile in lung tissues have largely been influenced by the paradoxical induction of the enzyme by environmental chemicals, as a result of continuous exposure to them. Other studies have attempted to explore a differential overexpression of CYP1A1 between tumor and normal cells, which can potentially add to the various applications of the enzyme in cancer pathology and treatment. Murray and colleagues have performed a series of studies on the expression of the extrahepatic CYPs in various tissues from normal and cancerous origin, in the absence of an inducer. The most significant finding of this research group was the identification of the enzyme CYP1B1 as a tumor marker, since the CYP1B1 protein was detected in a vast range of tumor tissues, irrespective of their oncogenic origin. Notably, however, the protein was absent in the corresponding normal samples [78]. Murray et al. have drawn similar associations regarding the differential overexpression of CYP1A1 in non-cancerous and cancerous tissues although this CYP1 isoform was shown to be present in a smaller range of tumors, compared to CYP1B1. CYP1A1 is present to a greater extent in malignant than in normal breast tissues, as determined by mRNA level expression [79-81], whereas in neoplastic mammary tissue oestradiol C-2 hydroxylase activity, which is a marker of CYP1A1 activity, was observed [82]. CYP1A enzymes were also present in a small percentage of non-neoplastic samples of oesophageal tissue, whereas in oesophageal carcinomas the enzymes were expressed in at least 60% of the samples [83]. Cytochromes P450 CYP1A were further detected in 68% of the urinary bladder tumors and their expression correlated with bladder tumor grade [84]. Such findings add to the well-established carcinogen-activating role of CYP1A1, since a higher expression of the enzyme would be expected in pre-malignant or malignant tissues due to the continuous exposure and subsequent metabolism by PAHs and related compounds.

Several studies have investigated the expression of CYP1A1 in human placenta cells because of the substrate specificity towards oestradiol and the potential carcinogenic and harmful effects to the developing fetus. Xenobiotic metabolism in the placenta is thought to be critical, particularly in the first trimester of pregnancy, whereas the activity of CYP-metabolizing enzymes declines in the second and third trimesters [85,86]. CYP1A1 has been found to be actively present in human placenta obtained from smokers, whereas in microsomes prepared from non-smokers the activity was considerably lower [87]. In the BeWo, the human choriocarcinoma cell line, CYP1A1, was readily induced as reported by Western immunoblotting and the EROD assay in the presence of PAHs (3-methylcholanthrene, 1,2-benzanthracene, α-napthoflavone), while in the absence of any inducer activity was minimal [88]. CYP1A1 activity in placenta tissues from subjects who were smokers has been well supported by other studies [89,90]. Elevated CYP1A1 activity in such tissues may contribute to several adverse birth outcomes, such as growth retardation and premature birth.

Further extrahepatic tissues in which CYP1A1 has been shown to be present include the intestine and the skin. It is becoming increasingly accepted that intestinal metabolism is a significant contributor to the hepatic metabolism of certain classes of xenobiotics [91]. CYP1A1 mRNA has been detected at low levels in the duodenum and jejunum of some donors [87,92,93]. Similar associations between smoking and CYP1A1 expression in the human duodenum were observed, compared to other extrahepatic tissues, i.e. CYP1A1 protein and activity were elevated in smokers [87]. CYP1A1 mRNA expression has also been detected in the skin, as well as in normal human keratinocytes. Finally recent studies have demonstrated the upregulation of CYP1A1 mRNA, protein and enzymatic activity in HUVECs, as well as human endothelial cells under shear stress, suggesting that an increased expression reflects an anti-atherogenic endothelial cell phenotype [94,95]. Constitutive CYP1A1 mRNA protein was also noted by immunostaining in endothelial cells [95].

It is generally accepted in the literature that as a result of exposure to environmental compounds with pro-mutagenic/pro-carcinogenic activity, the basal expression of CYP1A1 in extrahepatic tissues is linked to a great extent to the mechanism of chemical carcinogenesis. Nevertheless, further studies are required, in order to provide substantial insight to the exact mechanisms of CYP1A1 regulation in extrahepatic tissues.

### CYP1A1 polymorphisms

Several mutations in *CYP1A1 *have been found, corresponding to 15 different allelic variants http://www.imm.ki.se/CYPalleles. The first of these polymorphisms, CYP1A1* involves a thymidine to cytosine substitution at position 3801 of the 3' non-coding region downstream of the polyadenylation site [96]. Should the nucleotide change be found to be in the same position at the 3' non-coding region only, it is denoted CYP1A1*2B [97]. The second most commonly encountered *CYP1A1 *polymorphism involves an Adenine to Guanine base transition at position 2455A→G of codon 462 at exon 7, and is also known as CYP1A1*2B or Ile462Val due to the amino acid change [97]. If the amino acid is located in the haem-binding region, the corresponding variant is referred to as CYP1A1*2C [98].

Conventional theory regarding the effect of polymorphisms on CYP1A1 suggests that the variants affect the function of the enzyme by altering the level of gene expression or the mRNA stability, although the results in the literature appear to be contradictory [98]. For example, the Ile462Val polymorphism was found to confer increased levels of induced or basal CYP1A1 mRNA as the number of Val variants increased in one study, whereas in purified Escherichia Coli, no difference in the metabolism of benzo[*a*]pyrene between the Ile and Val variants was noted [99,100]. Similarly, a high activity or lack of correlation has been suggested with the 3801TC and Ile462Val polymorphisms regarding the activity of mutant enzymes in lymphocytes [101-104].

Generally, evidence regarding the association of genetic polymorphisms of CYP1A1 with cancer occurrence is conflicting, as some studies have concluded that there is increased susceptibility to cancer in the presence of polymorphic variants, whereas others have reported no relationship between the two [105,106]. Although a large body of experimental results points towards a positive association of CYP1A1 genetic polymorphisms and cancer occurrence, further investigation is required for such findings to be extrapolated successfully to human populations.

### Intracellular localization of CYP1A1

Cytochrome P450s are generally located in the endoplasmic reticulum and require the coenzyme NADPH reductase which catalyzes the two electron reduction of molecular O_2 _to H_2_O for their enzymatic activity. Early investigations showed that in addition to the ER localization, CYP1A1 is present in the mitochondria of liver tissue from rats pretreated with β-napthoflavone, a CYP1 inducer [107,108]. Subsequent studies revealed that CYP1A1 is present in both endoplasmic reticulum and mitochondrial inner membrane, depending on the tissue, animal age and inducer pretreatment. The enzyme is targeted to the mitochondria by proteolysis of cryptic MT-targeting signals, which remove a certain number of amino acids from the NH_2 _terminus, thus producing two alternative truncated isoforms of mitochondrial CYP1A1 (mt1A1). Mouse mt-Cyp1a1 shows distinct substrate specificity from the ER-associated form and metabolizes erythromycin as well as a number of psychotropic drugs, probably due to the presence of ferredoxin-1-reductase (FDX1) as a coenzyme, in place of the NADPH reductase found in the microsomal CYP1A1 [109]. A previous study supports the notion that it is the microsomal (mc-Cyp1a1) rather than the mitochondrial form of the enzyme (mtp-Cyp1a1) which contributes to B[*a*]P detoxication, by experiments in three knock-in C57B4/6J mice lines [110]. Cyp1a1(-/-) and Cyp1a1(mtp/mtp) mice showed striking toxicity following 18 days of daily oral B[*a*]P treatment, compared to wild-type and Cyp1a1 (mc/mc) mice, which were completely protected [110].

### CYP1A1 contribution to cancer prevention

The most significant line of evidence which contradicts the previously proposed carcinogen-activating role of CYP1A1, comes from Nebert and the generation of Cyp1a1 (-/-) knockout mice. C57BL/6J mice lacking the Cyp1a1 gene died within 30 days of 125 mg/kg/day oral B[*a*]P treatment, whereas Cyp1a1 (+/+) mice survived with no overt signs of toxicity [111]. In addition, the clearance rate of B[*a*]P in the Cyp1a1 (-/-) strain was 4 times slower as compared to Cyp1a1 (+/+) mice, while further pharmacokinetic experiments using TCDD and B[*a*]P suggested that clearance was almost exclusively dependent on inducible CYP1A1 and no other TCDD-B[*a*]P-inducible-metabolizing phase I enzyme [111]. B[*a*]P treatment had toxic effects in the immune system of Cyp1a1 (-/-) mice, while chemical depression of the bone marrow was noted at a dose as low as 1.25 mg/kg/day [111]. The data showed for the first time that this enzyme is more important in detoxication of B[*a*]P in the liver and intestine, rather than metabolic activation to its ultimate carcinogenic conversion products. From this initial observation the same group published a series of studies where Cyp1a1/1b1 (-/-) double knockout and Cyp1a1/1a2/1b1 (-/-) triple knockout mouse lines were generated from straightforward genetic crosses of Cyp1a1 (-/-), Cyp1b1 (-/-) and Cyp1a2 (-/-) knockout mice and tested for their ability to metabolize B[*a*]P. Compared with Cyp (+/+) wild-type mice, the Cyp1a1/1b1 (-/-) mice showed ~75 fold higher amounts of blood B[*a*]P after 5 days of feeding, demonstrating that the total body burden of B[*a*]P is independent of target organ damage [112]. The phenotype of this transgenic line revealed decreased spleen and thymus weights and increased liver weights [112]. Despite this discrepancy Cyp1a1/1b1 (-/-) and Cyp1b1 (-/-) mice displayed no significant difference in cellularity of the bone marrow following B[*a*]P treatment as opposed to Cyp1a1 (-/-) mice. B[*a*]P-DNA adduct patterns in Cyp1a1/1b1 (-/-) doubleknockout mice were proportionate to the relative degree of immunotoxicity and followed the order Cyp1a1 (-/-) >>>> Cyp1a1/1b1 (-/-) > Cyp (+/+) [112]. Thus although CYP1A1 contributes to detoxication, CYP1B1 expression in spleen and marrow is responsible for the metabolic activation of B[*a*]P, causing immune damage in mice which lack the Cyp1b1 gene [112]. The same "rescued" response following oral B[*a*]P treatment was observed in the triple knockout Cyp1a1/1a2/1b1 (-/-) mouse line, i.e. the absence of CYP1B1 in immune tissues is sufficient to avoid substantial bone marrow depression [113]. The phenotypic abnormalities of the triple knockout mouse line, which included a greater risk of embryolethality before gestational day 11, hermaphroditism and formation of cystic ovaries, were attributed to alterations in the production catabolism and/or formation of eicosanoids, bioactive mediators derived from arachidonic acid via ω-6 fatty acid synthesis and from eicosapentanoic acid via ω-3 fatty acids, which are involved in inflammation, innate immunity, angiogenesis and various other developmental processes [113]. Given that human and mouse CYP1A1 and CYP1A2 orthologs differ in the rates of metabolism of xenobiotics, Nebert and colleagues proceeded in the generation of a "humanized" hCYP1A1_CYP1A2_Cyp1a1/1a2 (-/-) mouse line. The successful insertion of both human CYP1A in place of the mouse Cyp1a genes returns the phenotype of the Cyp1a1/1a2 (-/-) double knockout line back to approximately that of the wild type, whereas the human CYP1A1 enzyme partially rescues the animal from oral B[*a*]P-induced immunosuppresion [114]. Although the hCYP1A1_CYP1A2_Cyp1a1/1a2 (-/-) mouse line offers unique opportunities for direct human CYP1A1 *in vivo *carcinogen metabolism studies caution should be taken as to whether results can successfully be extrapolated to human populations. For example, it is possible that the mouse relies more on the small intestine than the liver for B[*a*]P metabolism, whereas the opposite may be true for humans. Such organ differences regarding the metabolism of xenobiotics between the two species should be taken into account and further experiments are required to provide a full understanding of the use of this transgenic model in environmental toxicology and cancer pharmacology.

As mentioned earlier CYP1A1 enzyme expression is linked with important physiological and cell growth regulatory processes, through the involvement of AhR with multiple pathways other than the xenobiotic activating pathway. Recent experiments performed in lung tissue from mice which were AhR-null, suggest that AhR and CYP1A1 are subjected to an autoregulatory feedback loop, thus supporting the presence of an as yet unidentified endogenous ligand. Since the transient transfection of CV-1 cells with a CYP1A1 and an AhR expression vector led to a markedly reduced DRE-driven luciferase reporter activity, the authors of this study proposed that CYP1A1 in turn metabolizes the putative endogenous ligand, resulting in its inactivation and consequently in the attenuation of the AhR pathway [115]. As well as unraveling the precise contribution of this enigmatic orphan receptor in normal cellular metabolism, such evidence can add to the significance of excessive activation of AhR by xenobiotics and the identification of new molecular targets for therapeutic drug development [115].

**Figure 4:**
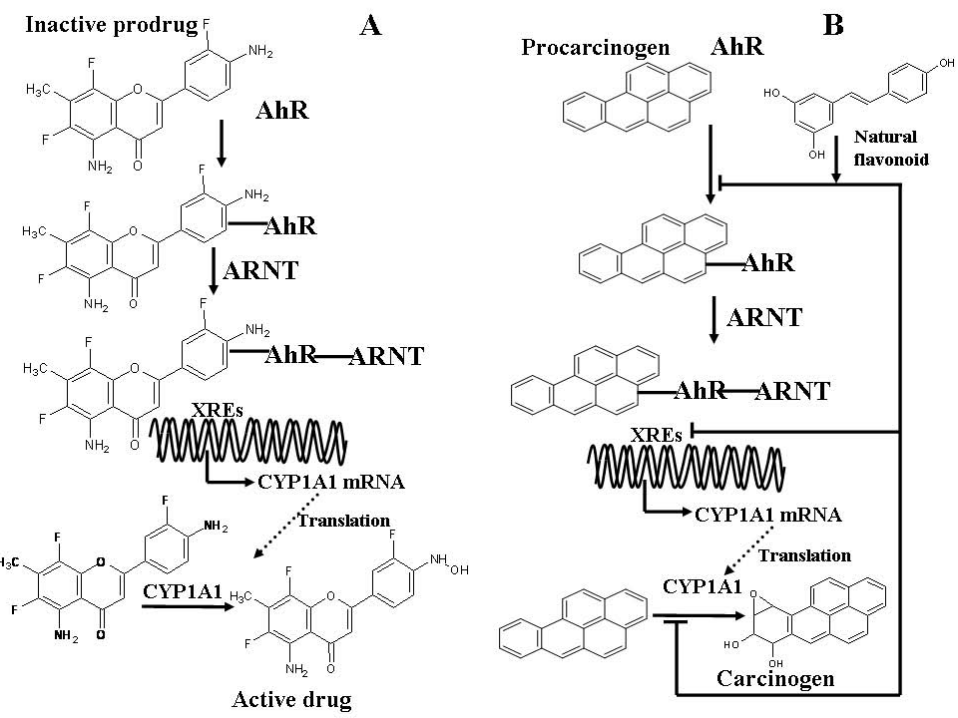
**The two different models for the contribution of CYP1A1 to chemoprevention**. (A) Molecular mechanism of aminoflavone AhR-induced CYP1A1 activation. It must be stressed that this mechanism of action is also likely to apply for certain dietary natural products, such as the flavonoid diosmetin, which induces CYP1A1 expression and is a substrate of the same enzyme [129,124,125]. (B) Chemopreventative action of natural products, such as the stilbene resveratrol, is based on the inhibition of benzo[*a*]pyrene binding to AhR, the binding of the AhR-ARNT-benzo[*a*]pyrene complex to xenobiotic response elements (XREs), and the inhibition of the formation of CYP1A1-mediated carcinogenic reactive intermediates, notably benzo[*a*]pyrene-7,8-diol-9,10-epoxide.

Regarding the pharmacological aspects of CYP1A1 xenobiotic-mediated metabolism accumulated evidence has corroborated the recent knockout mice findings, suggesting a cancer-protecting role of the enzyme by paradoxical activation of small synthetic molecules, as well as natural products present in the diet, to more antiproliferative agents. The synthetic compound Phortress or 2-(4-Amino-3-methylphenyl) benzothiazole is a CYP1A1-activated prodrug with potent *in vivo *activity in breast tumor xenografts [116]. Of note is that the natural AhR ligand indirubin and Phortress possess a very similar structure based on a benzimidazole ring. Nevertheless, this compound was not initially designed for the purpose of CYP1A1 induction. Initial studies suggested it had promising antitumour effects; however, its exact mechanism of action was elucidated in subsequent studies. Phortress, which entered early Phase I clinical trials in 2004, induces CYP1A1 in breast cancer sensitive cell lines, such as MCF-7, T-47D and IGROV (IC_50 _< 10 nM) and is further metabolized by CYP1A1 to reactive electrophillic species which results in DNA adduct formation [116-118]. This induction involves the binding of AhR to ARNT and translocation of the complex to the nucleus, as observed in the case of benzo[*a*]pyrene [119]. Another chemotherapeutic agent in this category, aminoflavone, also acts by the induction of CYP1A1 and its subsequent activation. Aminoflavone is active in MCF7 cell lines (IC_50 _= 0.1 nM) and MCF7 xenografts [120]. Again, AhR is required for CYP1A1 induction, rendering this compound 4,500-fold less active in MCF7 AhR-null mutant cells. Induced CYP1A1, in turn, converts aminoflavone to metabolites that form DNA protein crosslinks and cause DNA double-strand breaks, thereby inhibiting DNA synthesis in the S phase [121]. The molecular pathway of CYP1A1-mediated aminoflavone activation is shown in Figure 4A.

A small number of naturally occurring phytochemicals, which belong to the flavonoid subclass, have recently been identified as CYP1A1 substrates. The flavonoid eupatorin found in medicinal plants in South East Asia and South America, was recently shown to be converted to the structurally similar flavone cirsiliol, by an aromatic demethylation reaction catalysed by the enzymes CYP1A1 and CYP1B1 [122]. Eupatorin strongly inhibits the *in vitro *proliferation of MDA-MB-468 human breast cancer cells that express CYP1A1, but it is inactive in normal breast MCF-10A cells, devoid of any CYP1A1 activity [122]. The isoflavone daidzein, a component of soy beans, is a substrate for CYP1A1 as demonstrated by *in vitro *enzyme and cell-based assays [123]. Aromatic hydroxylation of daidzein at the 3' position enhances the antiproliferative activity of the compound in MCF-7 cells [123]. Similarly, the flavone diosmetin, present in olive leaves, is activated to the flavone luteolin, mainly by CYP1A1. CYP1A1-mediated metabolism of diosmetin in MDA-MB-468 and TCDD-induced MCF-7 cells increases its biological activity [124,125]. A recent study has reported that CYP1A1 has the highest rate of metabolism, compared to the hepatic CYPs 1A2, 3A4, 2C9 and 2D6, of fully methylated anticancer flavonoids such as tangeretin, a high constituent of orange peel [126]. The flavonols galangin and kaempferide have also been reported to be substrates of CYP1A1 [127]. Kaempferide is demethylated, while galangin is hydroxylated at position 4' to produce kaempferol another anticancer flavonoid found in black and green tea [128]. Of note is that the CYP1A1-catalyzed metabolism of dietary anticancer flavonoids produces compounds that also possess strong cancer preventative activity.

Certain flavonoids are capable of inducing CYP1A1 activity via the AhR in cancer cell line models [129-131]. The hypothesis that has been established by such findings follows the so-called blocking type of chemopreventative agent. Dietary constituents suppress cancer progression by inhibiting the CYP1A1-catalyzed metabolic activation and the CYP1A1 enzyme induction of carcinogens. The second occurs either by the blockage of AhR binding to the inducer or by the prevention of AhR-ARNT binding to XREs (Figure 4B) [132]. Although most of the compounds examined possess inhibitory activity against CYP1A1, the model does not adequately explain why CYP1A1 is induced and further inhibited. As the AhR-mediated induction of CYP1A1 is noted for a large amount of phytochemicals present in the diet, it can be assumed that the subsequent metabolism of these compounds is the end stage of this process, as has been noted for the procarcinogen benzo[*a*]pyrene [133]. More importantly, in *in vitro *or *in vivo *models where cell lines with a malignant phenotype are used, the inhibition of CYP1A1 ought not to alter the tumorigenic state of the cells, since they have already lost the ability to control their growth. In this sense, the action of dietary flavonoids and phytochemicals as CYP1A1 inhibitors is subject to further consideration. The induction of CYP1A1 in cancer cells by dietary compounds and their subsequent metabolism to more active agents is an alternative model that can explain the cancer preventative properties of these compounds in pharmacologically relevant concentrations [134]. In addition, many compounds that appear as CYP1A1 competitive inhibitors may well be CYP1A1 substrates.

## Conclusion

P450s are believed to have existed since the beginning of life over 3.5 billion years ago, but the P450s responsible for foreign compound metabolism appear to have arisen about 400 to 500 million years ago. It is believed that these enzymes were needed to metabolise and detoxify chemicals found in plants [135]. In the plant-animal "warfare" hypothesis, plants produce toxins to kill predators and animals evolve P450s to detoxify these toxins. As this process continues over millions of years new catalytic activities of P450s will develop. However, in the field of chemical carcinogenesis it is difficult to establish a clear relationship in which both the P450s and other foreign compound metabolizing enzymes would evolve for a beneficial purpose.

Although the majority of the studies have focused on the carcinogenic action of CYP1A1, it is recently becoming clear that this enzyme plays important roles in detoxication and chemoprevention, thus opposing the initially established concept, regarding its function in tumor progression. Moreover, extensive work on the molecular events governing the transcriptional activation of the *CYP1A1 *gene through the aryl hydrocarbon receptor has revealed the interplay of AhR with various cell signaling pathways, important in normal cell growth, homeostasis and development. The cross-talk of AhR with different signal transduction pathways is apparent. However, the precise mechanisms by which AhR ligands elicit toxic responses that may contribute to carcinogenesis still remain unclear. It was previously noted that this may be partially due to the majority of the studies coming from cancer-derived cell lines that have impaired cell cycle regulation and hence do not possess the full detoxication battery [34]. Studies in non-transformed cells or extrahepatic tissues have been proposed as better models of choice [34]. Utilization of these systems can unravel the exact mechanisms which regulate the expression of CYP1A1 in extrahepatic tissues, and offer insight into the contribution of the latter in cancer progression or prevention.

## List of abbreviations

CYP1A1: cytochrome P450 1A1; AhR: aryl hydrocarbon receptor; ARNT: aryl hydrocarbon nuclear translocator; PAH: polycyclic aromatic hydrocarbon; HIF-1: hypoxia inducible factor 1; bHLH: basic helix-loop-helix; PAS: per-ARNT-sim; HREs: hypoxia response elements; DREs: dioxin response elements, XREs: xenobiotic response elements; HSP90: heat-shock protein; XAP2: hepatitis B virus X-associated protein; AhRR: aryl hydrocarbon receptor repressor; SOCS-2: suppressor of cytokine signaling 2; VDAC2: voltage-dependent anion channel-selective protein 2; Cyp1a1(mc/mc): transgenic line carrying endoplasmic reticulum-targeted CYP1A1 protein; Cyp1a1(mtp/mtp): transgenic line carrying mitochondrial-targeted CYP1A1 via proteolysis; Cyp1a1(-/-): null mice lacking the Cyp1a1 gene; Cyp1a1/1b1(-/-): double knockout transgenic line lacking both Cyp1a1 and Cyp1b1 gene; Cyp1a1/1a2/1b1(-/-): triple knockout transgenic line lacking all three Cyp1 genes; hCYP1A1_CYP1A2_Cyp1a1/1a2(-/-): humanised transgenic line containing CYP1A genes in place of the mouse orthologs; EROD: 7-ethoxyresorufin-O-deethylase; ER: Estrogen receptor; GR: Glucocorticoid receptor; VEGF: Vascular endothelial growth factor; PDGF: Platelet-derived growth factor; Rb: Retinoblastoma; SRC-1: steroid receptor co-activator; NcoA2: nuclear co-activator 2; MAPK: mitogen activated protein kinase; ERK: extracellular signal-regulated kinase; JNK: jun N-terminal kinase; MEK: mitogen-activated extracellular signal regulated kinase.

## Competing interests

The authors declare that they have no competing interests.

## Authors' contributions

VPA participated in literature research and preparation of the first draft of the manuscript. AMT contributed substantially to the conception and design of the second draft of the manuscript. DAS made critical revisions for important intellectual content and has given approval of the final version to be published. All authors have read and approved the manuscript.

## Pre-publication history

The pre-publication history for this paper can be accessed here:

http://www.biomedcentral.com/1471-2407/9/187/prepub
